# Beschreibung und Anwendung primärer, sekundärer und tertiärer Präventionsmöglichkeiten in der Hals‑, Nasen- und Ohrenheilkunde

**DOI:** 10.1007/s00106-026-01746-0

**Published:** 2026-02-16

**Authors:** C. Schmit, M. Mayer, M. Santer, D. Dejaco, B. Hofauer

**Affiliations:** 1 Universitätsklinik für Hals‑, Nasen- und Ohrenheilkunde, Medizinische Universität Innsbruck, Anichstraße 35, 6020 Innsbruck, Österreich; 2 Klinik und Poliklinik für Hals‑, Nasen- und Ohrenheilkunde, Uniklinik Köln, Köln, Deutschland

**Keywords:** Risikofaktoren, Gesundheitliche Patientenaufklärung als Thema, Nichtübertragbare Krankheiten, Impfstoffe, Krankheitslast, Risk factors, Patient education as topic, Noncommunicable diseases, Vaccination, Disease burden

## Abstract

**Hintergrund:**

Prävention gewinnt in der modernen Medizin zunehmend an Bedeutung. Ziel ist die Vermeidung, Verzögerung oder Verminderung des Auftretens von Erkrankungen und damit eine Reduktion der Krankheitslast. Besonders in der Hals‑, Nasen- und Ohrenheilkunde bestehen vielfältige Ansatzpunkte zur Umsetzung präventiver Strategien. Der vorliegende Artikel bietet einen einführenden Überblick in das Leitthema der Prävention in der Hals-Nasen-Ohren-Heilkunde, während detaillierte Aspekte in den nachfolgenden Beiträgen dieser Sonderausgabe behandelt werden.

**Ziel der Arbeit:**

Der Beitrag gibt einen Überblick über etablierte und zukunftsorientierte Präventionsmaßnahmen in der HNO-Heilkunde und ordnet diese in das Konzept der Primär‑, Sekundär- und Tertiärprävention ein. Es erfolgte eine narrative Übersicht relevanter präventiver Ansätze anhand ausgewählter Beispiele aus der Literatur und klinischen Praxis.

**Ergebnisse:**

In der Primärprävention stehen Impfprogramme, Aufklärungsinitiativen zu Risikofaktoren wie Tabak, Alkohol und onkogenen Viren sowie Maßnahmen des Lärm- und Gehörschutzes im Vordergrund. Sekundärpräventive Strategien umfassen Screeninguntersuchungen, insbesondere das Neugeborenen-Hörscreening und audiometrische Testungen in Risikogruppen. Tertiärprävention findet sich in der onkologischen Nachsorge, der Hörrehabilitation und der Behandlung des chronischen Tinnitus durch multimodale Therapiekonzepte.

**Schlussfolgerung:**

Die konsequente Umsetzung präventiver Strategien auf allen Ebenen kann die Krankheitslast deutlich senken und die Lebensqualität der PatientInnen verbessern. Angesichts wachsender gesundheitlicher und ökonomischer Herausforderungen kommt der Prävention in der HNO-Heilkunde eine Schlüsselrolle in der Medizin des 21. Jahrhunderts zu.

## Abstract (en)

**Background:**

Prevention is gaining increasing importance in modern medicine. Its goal is to avoid, delay, or reduce the occurrence of diseases and thereby decrease the overall disease burden. In otorhinolaryngology, multiple opportunities exist for implementing preventive strategies. This article is intended to provide an introductory overview of the topic of prevention in otolaryngology, while detailed aspects will be addressed in subsequent articles of this special issue.

**Objective:**

This article provides an overview of established and emerging preventive measures in otorhinolaryngology and classifies them within the framework of primary, secondary, and tertiary prevention. A narrative review of relevant preventive approaches was conducted based on selected examples from current literature and clinical practice.

**Results:**

Primary prevention includes vaccination programs; public awareness campaigns on risk factors such as tobacco, alcohol, and oncogenic viruses; and occupational hearing protection. Secondary prevention comprises screening programs, particularly newborn hearing screening and audiometric testing in risk groups. Tertiary prevention involves oncologic follow-up care, hearing rehabilitation, and multimodal therapy for chronic tinnitus.

**Conclusion:**

Consistent implementation of preventive strategies at all levels can substantially reduce disease burden and improve patients’ quality of life. Given the increasing medical and economic challenges, prevention plays a key role in 21st-century otorhinolaryngology.

## Zentrale Konzepte

**Abb. 1:**
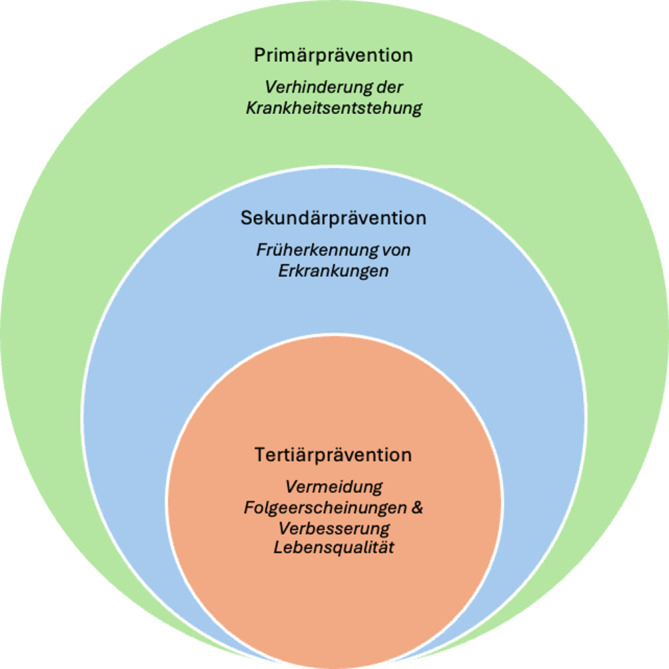
Schematische Darstellung der unterschiedlichen Präventionsebenen

Dem Begriff der Prävention kommt in der modernen Medizin eine kontinuierlich wachsende Bedeutung zu. Das Robert Koch-Institut (RKI) definiert Prävention als sämtliche Maßnahmen, die darauf abzielen, Erkrankungen zu vermeiden, hinauszuzögern oder deren Auftreten weniger wahrscheinlich zu machen – mit dem übergeordneten Ziel, die Krankheitslast in der Bevölkerung zu reduzieren. In Abhängigkeit von der zeitlichen Verortung im Krankheitsverlauf ergibt sich hieraus die heute gebräuchliche Einteilung in Primär‑, Sekundär- und Tertiärprävention, die ein grundlegendes Prinzip moderner medizinischer Versorgung darstellt (Abb. 1; [1, 2]).

Die HNO-Heilkunde weist ein besonders großes Potenzial für die Umsetzung präventiver Strategien auf

Neben den kontinuierlichen Fortschritten der kurativen Medizin bildet die Prävention einen essenziellen Pfeiler zur Bewältigung der gesundheitlichen Herausforderungen des 21. Jahrhunderts. Besonders im Kontext nicht übertragbarer Erkrankungen („noncommunicable diseases“, NCD) und der damit verbundenen hohen Krankheitslast kommt ihr eine zentrale Bedeutung zu [3].

Über die damit verbundene Reduktion der Krankheitslast hinaus eröffnet die Implementierung adäquater Strategien der Primär‑, Sekundär- und Tertiärprävention auch aus gesundheitsökonomischer Perspektive ein beträchtliches Potenzial [3].

Die HNO-Heilkunde weist durch ihre vielfältigen interdisziplinären Schnittstellen, das heterogene Patientenkollektiv sowie klar definierte Ansatzpunkte ein besonders großes Potenzial für die Umsetzung präventiver Strategien auf. Der vorliegende Beitrag versteht sich dabei ausdrücklich als einleitender Überblicksartikel zum Leitthema Prävention in der HNO-Heilkunde. Ziel ist es, zentrale Konzepte, Systematiken und exemplarische Anwendungsfelder darzustellen, ohne eine detaillierte Darstellung einzelner Maßnahmen anzustreben. Die vertiefte Auseinandersetzung mit spezifischen präventiven Aspekten erfolgt in den entsprechenden nachfolgenden Beiträgen dieses Themenhefts.

## Primärprävention

Die Primärprävention hat das vorrangige Ziel, das Entstehen von Krankheiten zu verhindern. Sie richtet sich gleichermaßen an gesunde Personen wie auch an definierte Risikogruppen [2]. Klassische Beispiele sind Impfprogramme, gesundheitliche Aufklärungs- und Informationskampagnen sowie Ernährungs- und Supplementierungsmaßnahmen [4]. Die folgenden Beispiele stellen exemplarisch dar, welche Ansatzpunkte für die Primärprävention in der HNO-Heilkunde bestehen. Eine detaillierte Betrachtung einzelner Interventionen erfolgt in den nachfolgenden Beiträgen.

### Impfkampagnen

Das bekannteste und am weitesten verbreitete Instrument der Primärprävention sind Impfungen [4]. Angesichts der auch in der Kopf-Hals-Onkologie zunehmend bedeutsamen Rolle onkogener Viren [5] ist die Hervorhebung von Impfprogrammen als spezifisches Präventionsinstrument im Fachgebiet der HNO-Heilkunde zunehmend relevant.

Neben den klassischen Risikofaktoren rücken in der Kopf-Hals-Onkologie zunehmend onkogene Viren in den Fokus

Kopf-Hals-Karzinome, bei denen es sich überwiegend um Plattenepithelkarzinome handelt, stellen weltweit die siebthäufigste Krebsentität dar [6]. Neben den klassischen Risikofaktoren, die im weiteren Verlauf erörtert werden, rücken zunehmend onkogene Viren, insbesondere das humane Papillomavirus (HPV), in den Fokus. Die inzwischen breit verfügbare HPV-Impfung stellt – ergänzt durch Aufklärungsmaßnahmen zur Reduktion der Übertragungswege – eine vielversprechende Möglichkeit zur Prävention HPV-assoziierter Kopf-Hals-Tumoren dar [5, 7–9]. Die großzügige Aufnahme von HPV-Impfstoffen in nationale Impfprogramme – auch über die derzeit definierten Altersgrenzen hinaus – stellt ein hochaktuelles Beispiel für eine mögliche Ausweitung der Primärprävention in der Hals‑, Nasen- und Ohrenheilkunde dar.

Eine detaillierte Auseinandersetzung mit diesem Thema erfolgt im eigens diesem Ansatz gewidmeten Beitrag.

### Aufklärungs- und Informationskampagnen

Ein weiteres zentrales Instrument der Primärprävention sind bevölkerungsgerichtete Aufklärungs- und Informationskampagnen zur Verhinderung von Krankheitsentstehung [4]. Neben den bereits genannten onkogenen Viren sind insbesondere Tabak- und Alkoholkonsum wesentliche Risikofaktoren für die Entstehung von Kopf-Hals-Karzinomen [6, 10, 11].

Die Ausweitung von Informationskampagnen zu onkogenen Viren ist daher eine unverzichtbare Ergänzung etablierter Impfprogramme. Solche Initiativen, die derzeit v. a. im schulischen Umfeld, zunehmend aber auch in der erwachsenen Allgemeinbevölkerung umgesetzt und gefördert werden [12], stellen durch den damit verbundenen Wissenszuwachs ein hocheffektives Instrument der Prävention dar [13, 14].

Obwohl die Rolle von Noxen in der Tumorentstehung wissenschaftlich eindeutig belegt [6, 10, 11] und in der Öffentlichkeit präsent ist, stellen Aufklärungs- und Informationskampagnen weiterhin ein zentrales Element der Präventionsstrategie dar. Ein prominentes Beispiel sind die seit 2016 in der Europäischen Union und in Deutschland verpflichtenden Warnhinweise und Bildkampagnen auf Tabakprodukten [15, 16]. Diese erreichen Risikogruppen direkt am Ort des Konsums und illustrieren damit anschaulich das Prinzip zielgerichteter Prävention. Eine Ausweitung dieser Präventionsstrategien durch die Einführung von Warnhinweisen auf alkoholischen Getränken, wie sie in mehreren europäischen Ländern bereits üblich ist, wird derzeit intensiv diskutiert [17].

Ernährungs- und Lifestyleberatung erscheint als Instrument primärpräventiver Strategien in der HNO-Heilkunde sinnvoll

Auch jenseits des onkologischen Spektrums der HNO-Heilkunde lassen sich potenzielle Ansatzpunkte für Informationskampagnen identifizieren. Ein Beispiel stellt die Ernährungs- und Lifestyleberatung dar, die insbesondere bei PatientInnen mit obstruktivem Schlafapnoesyndrom [18–20] oder gastroösophagealer und laryngealer Refluxerkrankung [21–23] zwar vorwiegend im Rahmen der Tertiärprävention Anwendung findet, jedoch auch als Instrument primärpräventiver Strategien sinnvoll erscheint.

### Hörschutz

Ein weiteres Beispiel für die Anwendung von Primärprävention in speziellen Risikogruppen stellen die im österreichischen ArbeitnehmerInnen-Schutzgesetz gesetzlich verankerten Maßnahmen zum Gehörschutz an Arbeitsstätten dar [24]. Die chronische Lärmschädigung, definiert als eine Schädigung im Bereich des Innenohrs durch langandauernde Einwirkung von Lärm mit einer Intensität von über 85 dB, stellt eine anerkannte Berufserkrankung dar [25]. Da als einzige therapeutische Maßnahme eine Hörrehabilitation mithilfe von Hörgeräten zur Verfügung steht, kommt der Prävention bei diesem Krankheitsbild eine zentrale Bedeutung zu [26]. Neben der Risikosensibilisierung ist insbesondere für exponierte Berufsgruppen die Bereitstellung geeigneter Schutzausrüstung ein klassisches Beispiel für die Umsetzung primärpräventiver Maßnahmen in der HNO-Heilkunde [26–28].

## Sekundärprävention

Die Sekundärprävention verfolgt das Ziel, Erkrankungen in einem frühen, noch asymptomatischen Stadium zu erkennen, um eine rechtzeitige Therapieeinleitung zu ermöglichen und dadurch die Krankheitslast zu reduzieren. Sie richtet sich somit an bereits erkrankte, jedoch asymptomatische Personen [2]. Klassische Beispiele sind entsprechende Screeningprogramme zur Früherkennung von Erkrankungen [4]. Dazu erfolgt eine exemplarische Darstellung entsprechender Strategien im Kontext der HNO-Heilkunde. Die detaillierte Darstellung ausgewählter sekundärpräventiver Maßnahmen ist einem gesonderten Beitrag vorbehalten.

### HNO-ärztliches Screening

Ein klassisches Beispiel für Sekundärprävention stellen sog. Screeninguntersuchungen dar. Ziel dieser Verfahren ist die frühzeitige Identifikation bislang klinisch nicht manifester Erkrankungen. Durch eine methodisch standardisierte Anwendung solcher Verfahren kann die Krankheitslast in definierten Risikogruppen reduziert werden, indem frühzeitig geeignete therapeutische Maßnahmen eingeleitet werden.

HNO-ärztliche Screeninguntersuchungen reichen von onkologischen Vorsorgeuntersuchungen bis hin zu schlafmedizinischen Messungen

Entsprechende Beispiele einer Implementierung von Sekundärprävention in Form von Screeninguntersuchungen in der HNO-Heilkunde sind vielfältig und reichen von onkologischen Vorsorgenuntersuchungen bis hin zu schlafmedizinischen Messungen in den entsprechenden Hochrisikogruppen.

### Hörscreening

Die bekannteste Anwendung von Sekundärprävention in der HNO-Heilkunde in Form von Screeningverfahren stellen audiometrische Untersuchungen dar, allen voran das Neugeborenen-Hörscreening.

Nach Angaben eines 2021 veröffentlichten Berichts der Weltgesundheitsorganisation (WHO) sind weltweit etwa 1,5 Mrd. Menschen von einer Form des Hörverlusts betroffen, mit steigender Tendenz. Angesichts der hohen globalen Krankheitslast sowie der zu erwartenden Zunahme unerkannter Fälle stellt die Etablierung standardisierter Screeninguntersuchungen eine zentrale präventive Maßnahme dar [29].

Neben dem bereits in dem vorherigen Abschnitt erwähnten beruflichen Risikogruppen erscheinen entsprechende methodische Screeninguntersuchungen, aufgrund der möglichen Auswirkungen einer nicht erkannten Presbyakusis insbesondere auf kognitive Funktionen, v. a. in der älteren Bevölkerung sinnvoll [30–32].

Darüber hinaus gehören Hörstörungen zu den häufigsten kongenitalen Behinderungen weltweit [33]. Eine frühzeitige Identifikation angeborener Hörstörungen durch das Neugeborenen-Hörscreening ermöglicht eine rasche Diagnostik und die zeitgerechte Einleitung individuell angepasster Rehabilitationsmaßnahmen [33]. Diese inzwischen flächendeckend etablierte Untersuchung stellt somit ein hochwirksames Instrument der Sekundärprävention dar.

Eine vertiefte Auseinandersetzung mit diesem Thema erfolgt im eigens diesem Ansatz gewidmeten Beitrag.

## Tertiärprävention

Die Tertiärprävention richtet sich an bereits erkrankte und symptomatische Personen mit dem Ziel, den Schweregrad der bestehenden Erkrankung zu verringern, Folgeerscheinungen zu vermeiden und die Lebensqualität zu verbessern [2]. Klassische Beispiele von Tertiärprävention entsprechen i. Allg. spezifischen, an die jeweilige Grunderkrankung angepassten Rehabilitationsmaßnahmen. Die nachfolgenden Beispiele verdeutlichen ausgewählte Ansatzpunkte tertiärpräventiver Strategien in der HNO-Heilkunde und verstehen sich ausdrücklich nicht als vollständige oder vertiefte Darstellung einzelner Maßnahmen.

### Onkologische Nachsorge

Ergänzend zu den bereits dargestellten Maßnahmen der Primär- und Sekundärprävention kommt auch den Strategien der Tertiärprävention in der Kopf-Hals-Onkologie eine zentrale Bedeutung zu.

Neben den im Rahmen der onkologischen Nachsorge in festgelegten zeitlichen Abständen etablierten klinischen und bildgebenden Kontrollen umfasst ein adäquates Nachsorgeschema auch weitere supportive und rehabilitative Interventionen, die teils spezifisch auf die jeweilige Tumorerkrankung und die individuellen Bedürfnisse der PatientInnen zugeschnitten sind. Exemplarisch hervorzuheben sind hierbei logopädische, diätologische, psychoonkologische sowie physiotherapeutische Maßnahmen [34].

Diese Maßnahmen verfolgen als direktes oder indirektes Ziel, tumorassoziierte Symptome zu lindern oder posttherapeutische Folgeerscheinungen zu vermeiden, und stellen damit essenzielle Bestandteile einer modernen onkologischen Tertiärprävention dar [35–38].

### Hörrehabilitation

Neben der Primärprävention im Sinne der Verhinderung der Entstehung von Hörstörungen sowie der Sekundärprävention durch Maßnahmen zur Früherkennung bereits manifester Hörstörungen stellt die Hörrehabilitation ein klassisches Beispiel für Tertiärprävention in der HNO-Heilkunde dar.

Eine adäquate Hörrehabilitation verfolgt unter anderem das Ziel der Reduktion bestehender Einschränkungen

Eine adäquate, an die jeweiligen Patientenbedürfnisse angepasste Hörrehabilitation verfolgt das Ziel der Reduktion bestehender Einschränkungen, der Wiederherstellung funktioneller Fähigkeiten sowie der Vermeidung sekundärer Folgen. Zu den entsprechenden Maßnahmen zählen sowohl die Versorgung mit individuell angepassten Hörgeräten als auch operative Verfahren bis hin zu Cochleaimplantation und begleitenden hörtherapeutischen Maßnahmen.

Die in zahlreichen Studien belegten positiven Effekte einer adäquaten Hörrehabilitation – insbesondere im Hinblick auf kognitive Funktionen und psychologische Komorbiditäten – verdeutlichen eindrucksvoll den nachhaltigen Nutzen für Patient:innen [30–32, 39].

### Chronischer Tinnitus

An das vorherige Thema anknüpfend lässt sich als weiteres Beispiel die Behandlung des chronischen Tinnitus anführen. Tinnitus, definiert als Wahrnehmung von Geräuschen ohne externe Schallquelle, betrifft etwa 15 % der Bevölkerung und ist mit erheblichen Komorbiditäten wie Depressionen, Angststörungen, Aufmerksamkeitsdefiziten und Schlafstörungen assoziiert [40–42].

Therapeutische Ansätze bei chronischem Tinnitus bestehen v. a. aus kognitiver Verhaltenstherapie, strukturiertem „Counselling“ sowie – sofern erforderlich – einer begleitenden Hörrehabilitation. Ziel dieser Maßnahmen ist primär die Reduktion des subjektiv erlebten Schweregrads, wodurch gleichzeitig mögliche Folgeerscheinungen reduziert und die Lebensqualität der PatientInnen nachhaltig verbessert werden können [43]. Damit stellt auch die Therapie des chronischen Tinnitus ein anschauliches Beispiel für die Anwendung von Tertiärprävention in der HNO-Heilkunde dar (Tab. 1).

**Tab. 1:** Präventionsstrategien in der HNO-Heilkunde – Zielgruppen und Beispiele

	Zielgruppe	Beispiele
Primärprävention	Gesunde Personen, spezifische Risikogruppen	Impfkampagnen (z. B. HPV, HiB, Pneumokokken usw.)
		Aufklärungs-, und Informationskampagnen (z. B. Bildkampagnen auf Tabakprodukten, Lifestyle- und Ernährungsberatung)
		Hörschutz am Arbeitsplatz
Sekundärprävention	Erkrankte, asymptomatische Personen	Screeninguntersuchung (z. B. NG-Hörscreening, onkologische Vorsorgeuntersuchungen bei positiver Noxenanamnese, schlafmedizinische Messungen bei Risikogruppen)
Tertiärprävention	Erkrankte, symptomatische Personen	Onkologische Nachsorge (inkl. logopädische, diätologische, psychoonkologische und physiotherapeutische Maßnahmen bei Kopf-Hals-Karzinomen)
		Rehabilitation (z. B. individuelle Hörrehabilitation, spezifische Verhaltenstherapie)

*HiB Haemophilus influenzae* Typ B, *HPV* humanes Papillomavirus, *NG-Hörscreening* Neugeborenen-Hörscreening

## Ausblick

Präventionsstrategien in der HNO-Heilkunde sind vielfältig und umfassen Maßnahmen der Primärprävention, wie Impfkampagnen und Aufklärungsprogramme, ebenso wie sekundärpräventive Screeningverfahren und rehabilitative Ansätze im Rahmen der Tertiärprävention.

Angesichts der durch konsequente Implementierung von Prävention erreichten Reduktion der Krankheitslast, nachhaltigen Verbesserung der Lebensqualität von Patient:innen und des erheblichen Potenzials aus gesundheitsökonomischer Sicht kommt der Prävention eine zentrale Rolle in der Medizin des 21. Jahrhunderts zu.

Als einleitender Beitrag stellt dieser Artikel die Prävention nicht erschöpfend dar, sondern verdeutlicht deren essenzielle Rolle in der HNO-Heilkunde und ermöglicht eine strukturierte Einordnung. Die folgenden Beiträge dieses Themenhefts greifen ausgewählte präventive Aspekte auf und vertiefen diese anhand spezifischer Krankheitsbilder, Patientengruppen und Interventionen.

## Fazit für die Praxis

- Prävention ist ein zentraler Bestandteil moderner HNO-Heilkunde und ergänzt kurative Therapien wirkungsvoll.
- Primärprävention: Impfprogramme und Aufklärungsmaßnahmen zu Risikofaktoren wie Tabak, Alkohol und onkogenen Viren sollten konsequent gefördert und ausgeweitet werden.
- Sekundärprävention: Früherkennungsprogramme, insbesondere das Neugeborenen-Hörscreening und regelmäßige Hörtestungen in Risikogruppen, sind essenziell zur Reduktion unerkannter Erkrankungen.
- Tertiärprävention: Eine strukturierte onkologische Nachsorge, adäquate Hörrehabilitation und multimodale Tinnitustherapie verbessern Lebensqualität und Langzeitprognose.
- Die Integration präventiver Strategien in den klinischen Alltag erfordert interdisziplinäre Zusammenarbeit und gezielte Patientenaufklärung.

## References

[1] Robert Koch Institut (2023) Themenschwerpunkt: Prävention 2023. https://www.rki.de/DE/Themen/Gesundheit-und-Gesellschaft/Praevention/themenschwerpunkt-praevention.html

[2] Kisling LA, Das JM. Prevention Strategies. StatPearls. Treasure Island (FL): StatPearls Publishing Copyright © 2025, StatPearls Publishing LLC.; 2025.

[3] Ärzteblatt D (2023) Prävention: Vorbeugen statt heilen. https://www.aerzteblatt.de/archiv/praevention-vorbeugen-statt-heilen-bab610c3-79b9-45ea-808c-5c3facc41f4e

[4] World, Health, Organization. Health promotion and disease prevention through population-based interventions, including action to address social determinants and health inequity n. d. Available from: https://www.emro.who.int/about-who/public-health-functions/health-promotion-disease-prevention.html.

[5] Roman BR, Aragones A (2021) Epidemiology and incidence of HPV-related cancers of the head and neck. J Surg Oncol 124(6):920–922 34558067 10.1002/jso.26687 PMC8552291

[6] Zygouras I, Leventakou D, Pouliakis A, Panagiotou S, Tsakogiannis D, Konstantopoulos G, et al. Human Papillomavirus 16 DNA Methylation Patterns and Investigation of Integration Status in Head and Neck Cancer Cases. Int J Mol Sci. 2023;24(19). 10.3390/ijms241914593 PMC10572864 37834041

[7] Drolet M, Bénard É, Pérez N, Brisson M (2019) Population-level impact and herd effects following the introduction of human papillomavirus vaccination programmes: updated systematic review and meta-analysis. Lancet 394(10197):497–509 31255301 10.1016/S0140-6736(19)30298-3 PMC7316527

[8] Chaturvedi AK, Graubard BI, Broutian T, Xiao W, Pickard RKL, Kahle L et al (2019) Prevalence of Oral HPV Infection in Unvaccinated Men and Women in the United States, 2009–2016. Jama 322(10):977–979 31503300 10.1001/jama.2019.10508 PMC6737522

[9] Merck (2020) FDA Approves Merck’s GARDASIL 9 for the Prevention of Certain HPV-Related Head and Neck Cancers. https://www.merck.com/news/fda-approves-mercks-gardasil-9-for-the-prevention-of-certain-hpv-related-head-and-neck-cancers/

[10] Zoschke IN, Bennis SL, Tang Y, Wilkerson JM, Stull CL, Nyitray AG et al (2025) The influence of tobacco use, hazardous drinking, and other risk factors on HPV-associated oropharyngeal cancer risk and screening perceptions among gay and bisexual men: a cross-sectional study. BMC Oral Health 25(1):462 40159495 10.1186/s12903-025-05774-0 PMC11955142

[11] Zygogianni AG, Kyrgias G, Karakitsos P, Psyrri A, Kouvaris J, Kelekis N et al (2011) Oral squamous cell cancer: early detection and the role of alcohol and smoking. Head Neck Oncol 3:2 21211041 10.1186/1758-3284-3-2 PMC3022893

[12] MSD. Der HPV-Förderpreis zur Aufklärung über Humane Papillomaviren 2025. Available from: https://www.msd.at/de/hpv-forderpreis/.

[13] Zomordi G, Moradi M, Hasanzadeh M, Ghavami V (2022) The effect of education based on the theory of planned behavior on the intention of vaccination against human papillomavirus in female students: A controlled educational trial. J Educ Health Promot 11:237–237 36177425 10.4103/jehp.jehp_1145_21 PMC9514255

[14] Ziaee A, Ziaee M, Asghari A, Elhamirad S, Azarkar G (2024) Unpacking HPV Stigma: Assessing Healthcare Workers’ Knowledge and Stigma Towards HPV While Exploring the Connection Between the Two. J Med Educ Curric Dev 11:23821205241260596 38846082 10.1177/23821205241260596 PMC11155363

[15] Deutscher, Bundestag (2016) Bundestag verabschiedet neues Tabakgesetz 2016. https://www.bundestag.de/webarchiv/textarchiv/2016/kw08-de-tabakprodukte-409518

[16] DK (ed) (2009) Ein Bild sagt mehr als tausend Worte: Kombinierte Warnhinweise aus Bild und Text auf Tabakprodukten. Deutsches Krebsforschungszentrum, Heidelberg

[17] World, Health, Organization (2025) Alkohol-Etiketten sollten auf Krebsrisiko hinweisen, so ein neuer Bericht von WHO/Europa 2025. https://www.who.int/europe/de/news/item/14-02-2025-alcohol-labels-should-warn-of-cancer-risk--says-new-who-europe-report

[18] Mou J, Zhou H, Huang S, Feng Z (2024) The impact of comprehensive healthy lifestyles on obstructive sleep apnea and the mediating role of BMI: insights from NHANES 2005-2008 and 2015-2018. BMC Pulm Med 24(1):601 39633317 10.1186/s12890-024-03404-z PMC11619612

[19] Duan X, Huang J, Zheng M, Zhao W, Lao L, Li H et al (2022) Association of healthy lifestyle with risk of obstructive sleep apnea: a cross-sectional study. BMC Pulm Med 22(1):33–33 35016643 10.1186/s12890-021-01818-7 PMC8751297

[20] Zheng YB, Huang YT, Gong YM, Li MZ, Zeng N, Wu SL et al (2024) Association of lifestyle with sleep health in general population in China: a cross-sectional study. Transl Psychiatry 14(1):320 39098892 10.1038/s41398-024-03002-x PMC11298538

[21] Zhang T, Lu M, Li Z, Zheng J, Cao J, Zhou Y et al (2025) Diet, genetic factors, and the risk of gastroesophageal reflux disease, Barrett’s esophagus and esophageal adenocarcinoma. Food Funct 16(18):7082–7093 40878106 10.1039/d5fo00369e

[22] Ma C, Xu Y, Zhang L, Li D, Long Z, Hu F (2025) Sweetened beverage intake and incident gastroesophageal reflux disease in a prospective cohort study. Eur J Nutr 64(5):219–219 40498344 10.1007/s00394-025-03707-9

[23] Zhang M, Hou ZK, Huang ZB, Chen XL, Liu FB (2021) Dietary and Lifestyle Factors Related to Gastroesophageal Reflux Disease: A Systematic Review. Ther Clin Risk Manag 17:305–323 33883899 10.2147/TCRM.S296680 PMC8055252

[24] Umwelt, Bundesamt (2021) Gehörschäden. https://www.umweltbundesamt.de/themen/laerm/laermwirkungen/gehoerschaeden#wie-entstehen-gehorschaden

[25] (BAuA) BfAuA. Liste der Berufskrankheiten. Dortmund; 2025. Contract No.: 19.09.2025.

[26] Le TN, Straatman LV, Lea J, Westerberg B (2017) Current insights in noise-induced hearing loss: a literature review of the underlying mechanism, pathophysiology, asymmetry, and management options. J Otolaryngol Head Neck Surg 46(1):41 28535812 10.1186/s40463-017-0219-x PMC5442866

[27] Neufeld A, Westerberg BD, Nabi S, Bryce G, Bureau Y (2011) Prospective, randomized controlled assessment of the short- and long-term efficacy of a hearing conservation education program in Canadian elementary school children. Laryngoscope 121(1):176–181 21120832 10.1002/lary.21185

[28] Verbeek JH, Kateman E, Morata TC, Dreschler WA, Mischke C (2014) Interventions to prevent occupational noise-induced hearing loss: a Cochrane systematic review. Int J Audiol 53(Suppl 2(0 2)):S84–S96 24564697 10.3109/14992027.2013.857436 PMC4678960

[29] Organization WH. World report on hearing: executive summary. Geneva; 2021.

[30] Rutherford BR, Brewster K, Golub JS, Kim AH, Roose SP (2018) Sensation and Psychiatry: Linking Age-Related Hearing Loss to Late-Life Depression and Cognitive Decline. Am J Psychiatry 175(3):215–224 29202654 10.1176/appi.ajp.2017.17040423 PMC5849471

[31] Lin FR, Yaffe K, Xia J, Xue QL, Harris TB, Purchase-Helzner E et al (2013) Hearing loss and cognitive decline in older adults. JAMA Intern Med 173(4):293–299 23337978 10.1001/jamainternmed.2013.1868 PMC3869227

[32] Lin FR, Metter EJ, O’Brien RJ, Resnick SM, Zonderman AB, Ferrucci L (2011) Hearing loss and incident dementia. Arch Neurol 68(2):214–220 21320988 10.1001/archneurol.2010.362 PMC3277836

[33] Wen C, Zhao X, Li Y, Yu Y, Cheng X, Li X et al (2022) A systematic review of newborn and childhood hearing screening around the world: comparison and quality assessment of guidelines. BMC Pediatr 22(1):160–160 35351033 10.1186/s12887-022-03234-0 PMC8962144

[34] (NCCN) NCCN (2025) NCCN Clinical Practice Guidelines in Oncology (NCCN Guidelines®): Head and Neck Cancers: National Comprehensive Cancer Network. https://www.nccn.org/professionals/physician_gls/pdf/head-and-neck.pdf

[35] Govender R, Gilbody N, Simson G, Haag R, Robertson C, Stuart E (2024) Post-Radiotherapy Dysphagia in Head and Neck Cancer: Current Management by Speech-Language Pathologists. Curr Treat Options Oncol 25(6):703–718 38691257 10.1007/s11864-024-01198-0 PMC11222272

[36] Prevost V, Joubert C, Heutte N, Babin E (2014) Assessment of nutritional status and quality of life in patients treated for head and neck cancer. Eur Ann Otorhinolaryngol Head Neck Dis 131(2):113–120 24657191 10.1016/j.anorl.2013.06.007

[37] Smith BG, Lewin JS (2010) Lymphedema management in head and neck cancer. Curr Opin Otolaryngol Head Neck Surg 18(3):153–158 20463478 10.1097/MOO.0b013e32833aac21 PMC4111092

[38] M H (2023) Psychosocial Oncology: Optimizing Outcomes through Interdisciplinary Care in Head and Neck Oncology. Curr Oncol 30:6859–6861 37504361 10.3390/curroncol30070501 PMC10378608

[39] Castiglione A, Benatti A, Velardita C, Favaro D, Padoan E, Severi D et al (2016) Aging, Cognitive Decline and Hearing Loss: Effects of Auditory Rehabilitation and Training with Hearing Aids and Cochlear Implants on Cognitive Function and Depression among Older Adults. Audiol Neurootol 21(Suppl 1):21–28 27806352 10.1159/000448350

[40] Kleinjung T, Peter N, Schecklmann M, Langguth B (2024) The Current State of Tinnitus Diagnosis and Treatment: a Multidisciplinary Expert Perspective. J Assoc Res Otolaryngol 25(5):413–425 39138756 10.1007/s10162-024-00960-3 PMC11528090

[41] Czornik M, Malekshahi A, Mahmoud W, Wolpert S, Birbaumer N (2022) Psychophysiological treatment of chronic tinnitus: A review. Clin Psychol Psychother 29(4):1236–1253 34994043 10.1002/cpp.2708

[42] Langguth B, Shiao AS, Lai JT, Chi TS, Weber F, Schecklmann M et al (2023) Tinnitus and treatment-resistant depression. Prog Brain Res 281:131–147 37806713 10.1016/bs.pbr.2023.01.001

[43] Dalrymple SN, Lewis SH, Philman S (2021) Tinnitus: Diagnosis and Management. Am Fam Physician 103(11):663–671 34060792

